# Embryonal Testicular Cancer with Duodenal Metastasis: Could Nausea and Vomiting be Alarm Symptoms?

**DOI:** 10.5005/jp-journals-10018-1200

**Published:** 2016-12-01

**Authors:** Mustafa Emre Duygulu, Mustafa Kaymazli, Ibrahim Goren, Beytullah Yildirim, Yurdanur Sullu, Mehmet Selim Nural, Ahmet Bektas

**Affiliations:** 1 Department of Internal Medicine, Ondokuz Mayis University, Samsun, Turkey; 2 Department of Gastroenterology, Ondokuz Mayis University, Samsun, Turkey; 3 Department of Pathology, Ondokuz Mayis University, Samsun, Turkey; 4 Department of Radiology, Ondokuz Mayis University, Samsun, Turkey

**Keywords:** Duodenal metastasis, Testicular cancer, Vomiting.

## Abstract

**Aim:**

Duodenal metastasis of testicular cancer is an uncommon condition in clinical practice. Here, we have reported a case of this nature.

**Background:**

Testicular cancers are among the most seen cancer types among young men. Metastasis of testicular cancer generally occurs through hematogenous and lymphatic drainage. Gastrointestinal (GI) metastasis of testicular cancer has been reported rarely.

**Case report:**

A duodenal mass was seen in esophagogastroduodenoscopic examination in a man who was admitted into hospital for medical treatment of resistant nausea and vomiting. He was previously diagnosed with testicular cancer. Computed tomography (CT) views were compatible with primary duodenal tumor. The duodenal mass was compatible with germ cell neoplasm metastasis. He received chemotherapy regime which includes cisplatin, paclitaxel, and ifosfamid. Nausea and vomiting symptoms decreased and metastatic mass and lymph nodes were regressed.

**Conclusion:**

Duodenum metastasis of testicular cancer can be treated with a chemotherapy regimen, and patients can improve radiologically and symptomatically without the need of any surgery. Physicians should keep in mind that GI metastasis of testicular cancer may present with nausea and vomiting symptoms.

**How to cite this article:**

Duygulu ME, Kaymazli M, Goren I, Yildirim B, Sullu Y, Nural MS, Bektas A. Embryonal Testicular Cancer with Duodenal Metastasis: Could Nausea and Vomiting be Alarm Symptoms? Euroasian J Hepato-Gastroenterol 2016;6(2):198-201.

## INTRODUCTION

Testicular cancer is one of the most commonly seen solid tumors among young men.^1^ Metastasis of testicular cancer generally occurs through hematogenous and lymphatic drainage pathways.^2^ Gastrointestinal (GI) metastasis of testicular cancer has been reported very rarely.^2^ Here, we have reported that a duodenal mass was seen in esophagogastroduodenoscopic examination of a man with complaints of nausea and vomiting. Biopsy result of the duodenal mass was compatible with the metastasis of a germ cell tumor.

## CASE REPORT

**Figs 1A to F:**
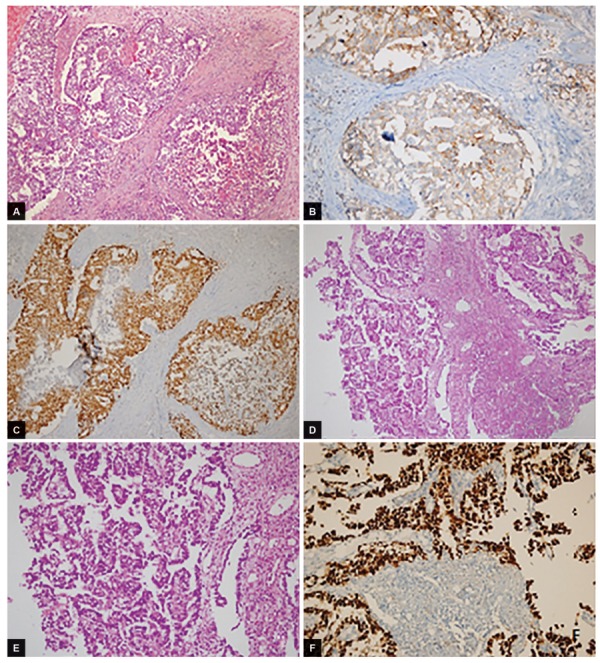
(A) Histopathologic evaluation of the specimen revealed that the lesion is a metastatic mass originated from the testicular germ cell tumor. Embriyonal carcinoma of testes ×200; hematoxylin and eosin stain; (B) CD30 stain; (C) Oct-3/4 stain; (D) hematoxylin and eosin stain ×100; (E) hematoxylin and eosin stain ×200; and (F) Oct-3/4 stain ×200

**Figs 2A to D:**
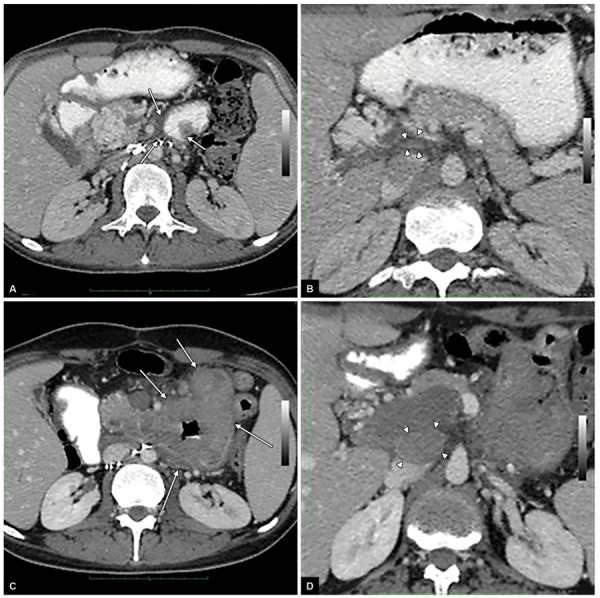
Abdominal CT applied before treatment showed metastatic duodenal mass on the right (C and D arrows). After treatment with a chemotherapy regimen composed of cisplatin, paclitaxel, and ifosfamide, a follow-up CT examinations revealed that there was a prominent regression in the metastatic mass and the enlarged lymph nodes seen on the left (A and B arrows)

A 22-year-old male patient was diagnosed with a testicular cancer, and right orchiectomy was performed. Histopathologic examination revealed the diagnosis as a nonseminamatosis germ cell neoplasia. He was treated with a chemotherapy regimen composed of cisplatin, paclitaxel, and ifosfamide. The patient attended our clinic with intractable nausea and vomiting complaints. The pathological specimens of the patient were reevaluated. The diagnosis was considered as an embryonal carcinoma after this reevaluation. A duodenal mass was seen on the esophagogastroduodenoscopic examination. Although computed tomography (CT) views were similar to primary duodenal tumor, histopathologic evaluation of the specimen revealed that the lesion is a metastatic mass originated from the testicular germ cell tumor (Figs 1A to F). After treatment with a chemotherapy regimen composed of cisplatin, paclitaxel, and ifosfamide, a follow-up CT examinations revealed that there was regressions of metastatic mass and in the enlarged lymph nodes (Figs 2A to D). After the treatment, nausea and vomiting complaints were also improved.

## DISCUSSION

Testicular cancers compose a small part of all male malignancies. Incidence of the testicular cancer differs between geographic areas. High incidences have been reported in Europe, and low incidences have been reported in Africa and Asia.^1^ Most of them are germ cell tumors. Germ cell tumors in testis have been divided into two groups: Seminoma tumors and nonseminoma tumors. Twenty percent of the testicular carcinoma cases are embryonal cancer, and it is a member of nonseminomatous germ cell tumors.^3^ Seminomas are generally limited to testis, and they have a higher sensitivity to radiotherapy.^4^ Metastatic spread of testicular cancer may occur through both of the hematologic and the lymphatic drainage systems. Gastrointestinal tract metastases are seen in less than 5% of germ cell tumor cases. In metastatic cases, embryonal carcinomas are more commonly seen compared to the other types.^5^ Gastrointestinal metastasis is seen in nonseminamatos tumors more commonly than in other types of testicular cancer. This metastasis occurs through the direct spread after the enlargement of retroperitoneal lymph nodes which serves the drainage of testicular lymphatic fluids.^2,5,7^ Metastases into the intestinal retroperitoneal area are seen more commonly than the ones that spread into the other retroperitoneal areas, and this is because of the lymphatic drainage pathway of testes.^2^ It has been shown that modern chemotherapy regimens are effective for the treatment of metastatic testicular tumors.^6,8^ It is known that some cancers could spread into the small intestine. These cancers are mainly melanoma, lung, adrenal, testicular, ovarian, gastric, large intestine, pancreatic, uterine, cervix, liver, and kidney cancers.^9^ Besides the symptoms of anemia and stomach pain, bleeding, perforation, obstruction, and intussusceptions can also be seen in the cases of testicular tumor GI metastasis.^7,10^ The patient presented here attended our hospital for treatment of resistant nausea and vomiting complaints. The patient had already been diagnosed with embryonic testicular cancer. Esophagogastroduodenoscopic examination revealed that there were a duodenal mass in the GI tract, and after taking a biopsy, histopathological examination of the mass revealed a diagnosis of germ cell tumor metastasis to the duodenum.

Duodenum metastasis of testicular cancer could be seen rarely, and it can be treated with a chemotherapy regimen which is composed of cisplatin, paclitaxel, and ifosfamide. After this treatment, patients can improve radiologically and symptomatically without the need of any surgery.

Before turning into any advanced stage, such as perforation or obstruction, physicians should consider the possibility of duodenal metastasis with nausea and vomiting complaints in the young patients of germ cell tumor.

## CONCLUSION

Physicians should keep in mind that GI metastasis of testicular cancer may present with nausea and vomiting symptoms and this condition can be treated without need of any surgery.
